# Anti-*Aspergillus* activities of olorofim at sub-MIC levels during early-stage growth

**DOI:** 10.1128/spectrum.03304-23

**Published:** 2024-02-05

**Authors:** Alexander Kühbacher, Mike Birch, Jason D. Oliver, Fabio Gsaller

**Affiliations:** 1 Institute of Molecular Biology, Biocenter, Medical University of Innsbruck, Innsbruck, Austria; 2 F2G Ltd., Manchester, United Kingdom; Universidade do Minho, Braga, Portugal

**Keywords:** olorofim, voriconazole, *Aspergillus*, sub-MIC, high-throughput microscopy, antifungal susceptibility testing

## Abstract

Olorofim, the first member of the novel class of antifungal drugs, the orotomides, shows promising anti-*Aspergillus* activity and is currently in phase III clinical development. Using high-throughput microscopy, we monitored olorofim’s antifungal potential at sub-minimum inhibitory concentration (MIC) levels with a focus on early-stage growth. Unlike voriconazole, olorofim showed significant growth inhibitory activities against three main pathogenic *Aspergillus* species, *Aspergillus fumigatus*, *Aspergillus flavus*, and *Aspergillus niger*, at concentrations >100,000-fold below its MIC.

**IMPORTANCE:**

Among antifungal compounds in clinical development for systemic disease, the orotomide olorofim is one of only two that target a completely new mechanism of action. Olorofim is highly potent against pathogenic *Aspergillus* species including cryptic species that frequently show increased resistance to current agents. In this study, our primary focus was on evaluating in detail the inhibitory activity of voriconazole and olorofim against different pathogenic *Aspergillus* species employing high-throughput microscopy. Compared to standardized, less-sensitive visual assessment-based methods, microscopy-assisted growth monitoring allowed us to detect sub-MIC drug concentration ranges with significant inhibitory activity at early-stage growth. This revealed that olorofim exerts growth inhibition at concentrations that are several magnitudes below those of voriconazole.

## OBSERVATION

A large proportion of deaths caused by human fungal pathogens are related to infections caused by species of the genus *Aspergillus* (1). Up to today, the repertoire of antifungal agents for the treatment of aspergillosis is limited to three major drug classes with azole antifungals being the first-line treatment of choice (2, 3). Over the past years, the worldwide distribution and emergence of azole resistance have raised concerns about their future clinical use (4, 5), which stresses the demand for novel, potent antifungal agents with novel mechanisms of action. Several antifungals with promising anti-*Aspergillus* activities are currently in clinical development including olorofim (previously named F901318) (6–9), which is the first representative of the new antifungal drug class, the orotomides, that entered clinical phase III in 2022 (https://clinicaltrials.gov/ct2/show/NCT05101187). Olorofim inhibits dihydroorotate dehydrogenase, a crucial enzyme in the *de novo* biosynthesis of pyrimidines (8). Previous work demonstrated that olorofim was highly active against major pathogenic *Aspergillus* species including *Aspergillus fumigatus*, *Aspergillus flavus*, and *Aspergillus niger* with mean minimum inhibitory concentration (MIC) levels <0.031 µg/mL. In addition to *Aspergillus* spp., its spectrum of activity comprises various further filamentous and pathogenic dimorphic fungi (8–10).

In this work, we assessed in detail the antifungal activity of olorofim at early-stage growth and compared it to that of the first-line treatment agent voriconazole (2) with a focus on its inhibitory potential at sub-MIC level. For this, we carried out high-throughput microscopy to detect growth-based confluence values using the IncuCyte S3 Live-Cell Analysis System (Essen Bioscience Inc., Ann Arbor, MI, USA), which proved to be a powerful instrument for the detection of antifungal effects during the early growth (11). The analyses comprised three major pathogenic *Aspergillus* species *A. fumigatus* (ATCC 204305), *A. flavus* (ATCC 204304), and *A. niger* (ATCC 9029). GraphPad Prism 9 software (Dotmatics, Boston, MA, USA) was used to analyze and display results. All experiments were carried out in triplicate. While cultivation steps were performed following the broth microdilution reference method of the European Committee on Antimicrobial Susceptibility Testing (12) using an inoculum of 1 × 10^5^ conidia/mL, the monitored concentration ranges for voriconazole were 4 to 0.008 mg/L and 0.2 to 3.73 × 10^−10^ mg/L for olorofim, as the drug showed activity far below its MIC. For microscopic analysis, strains were incubated for 12 h at 37°C. Growth was monitored in the absence (no drug control) and presence of serial dilutions of each drug, which allowed the determination of the inhibitory effects of each drug concentration. Percent growth reduction was calculated by normalizing the measured confluence values to the respective no-drug control.

**Fig 1:**
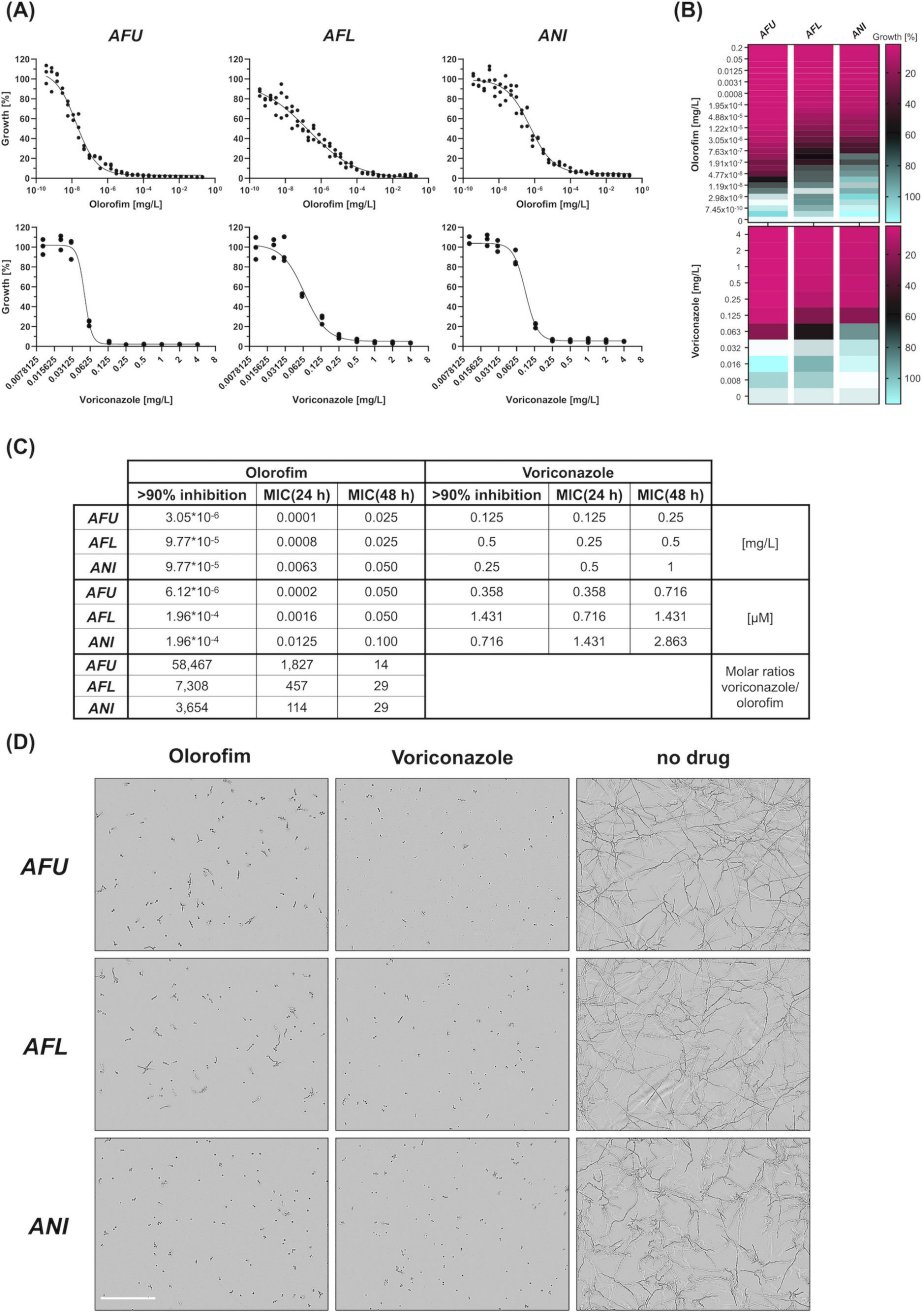
Antifungal activity of olorofim and voriconazole against pathogenic *Aspergillus* species. (**A**) Growth inhibition curves of *A. fumigatus* (*AFU*), *A. flavus* (*AFL*), and *A. niger* (*ANI*) treated with different concentrations of olorofim (log10 scale) and voriconazole. (**B**) Heatmap illustrating relative growth of strains in the presence of serial dilutions of each drug. (**C**) For each strain and each drug, the lowest concentrations that led to >90% growth inhibition were determined. Drug concentrations are given in milligrams per liter (mg/L) as well as micromolar (µM), and the latter was used to compare the activities of olorofim and voriconazole (molar ratio). (**D**) Microscopic images displaying strains at >90% growth inhibition and the no-drug control. If not otherwise indicated, growth was analyzed after 12 h incubation at 37°C. Scale bar: 200 µm.

Statistically significant (*P* < 0.05) growth reduction of all species was achieved at a concentration of 0.063 mg/L for voriconazole, whereby the highest growth reduction at this drug level was observed for *A. fumigatus* (78%) followed by *A. flavus* (46%) and *A. niger* (19%) (Table 1 displays growth rates as a % of no drug control; further details are provided in supplemental data 1). For olorofim concentrations, several magnitudes below its detected MIC significantly inhibited growth of each species; i.e., 2.98 × 10^−9^ mg/L were required for *A. fumigatus* (12%) and 1.19 × 10^−8^ mg/L and 1.91 × 10^−7^ mg/L, respectively, for *A. flavus* (33%) and *A. niger* (38%). Due to the small activity range of voriconazole compared to olorofim (up to 4 and 23 serial dilutions below the MIC, respectively), this would not allow an adequate comparison of the two drugs’ activities. Therefore, we further determined the smallest concentrations of each compound that led to severe growth reduction (>90%) (Fig. 1). This was achieved with voriconazole at 0.125 mg/L for *A. fumigatus* (96.0%), 0.5 mg/L for *A. flavus* (93.3%), and 0.25 mg/L for *A. niger* (94.3%). The minimal olorofim levels required were 3.05 × 10^−6^ mg/L for *A. fumigatus* (93.3%) and 9.77 × 10^−5^ mg/L for both *A. flavus* and *A. niger* (91.4% and 91.6%, respectively). Considering similar growth inhibition at these concentrations (<5% difference; 91.4–96.0%) with both drugs for all species, we further determined fold differences (molar ratios) employing the respective voriconazole and olorofim concentrations. The molar concentration ratios (voriconazole vs olorofim) were 58,467 for *A. fumigatus* (0.358 µM vs 6.12 × 10^−6^ µM), 7,308 for *A. flavus* (1.431 µM vs 1.96 × 10^−4^ µM), and 3,654 for *A. niger* (0.716 µM vs 1.96 × 10^−4^ µM), which further emphasizes the large differences in the activities of the two drugs on early-stage growth inhibition.

**TABLE 1:** Relative growth rates in the presence of serial dilutions olorofim and voriconazole^*a*^

Drug (mg/L)	Growth ± SD (%)								
	*A. fumigatus*			*A. flavus*			*A. niger*		
Olorofim									
2.00E-01	1.2 ± 0.13			2.3 ± 0.45			3.3 ± 0.68		
1.00E-01	1 ± 0.02			3 ± 1.7			3.2 ± 0.81		
5.00E-02	1.3 ± 0.02			2.9 ± 1.17			3.7 ± 0.98		
2.50E-02	1.4 ± 0.13			2.9 ± 0.42			3.4 ± 1.33		
1.25E-02	1.7 ± 0.12			2.7 ± 0.17			4 ± 0.47		
6.25E-03	1.8 ± 0.26			2.3 ± 0.65			3.8 ± 0.82		
3.13E-03	1.9 ± 0.28			2.9 ± 0.04			3.4 ± 0.04		
1.56E-03	2 ± 0.37			3.2 ± 1.02			4.8 ± 0.45		
7.81E-04	1.8 ± 0.37			5 ± 1.9			5 ± 0.65		
3.91E-04	2.2 ± 0.1			5.3 ± 1.29			7.1 ± 0.78		
1.95E-04	2.6 ± 0.72			5.8 ± 0.04			6.6 ± 1.2		
9.77E-05	2.6 ± 0.8			8.6 ± 0.42			8.4 ± 2.76		
4.88E-05	3.2 ± 0.94			13.7 ± 2.86			12.7 ± 1.33		
2.44E-05	3.3 ± 0.61			10.9 ± 0.47			14.9 ± 0.75		
1.22E-05	4.2 ± 1.31			18.3 ± 4.44			13.9 ± 0.85		
6.10E-06	5.8 ± 1.12			25.6 ± 4.32			17.7 ± 0.44		
3.05E-06	6.7 ± 2.01			28.4 ± 9.52			26 ± 5.39		
1.53E-06	12.3 ± 3.1			35.7 ± 4.35			36.1 ± 8.91		
7.63E-07	14.6 ± 0.62			40.2 ± 5.43			39 ± 3.84		
3.81E-07	16.7 ± 5.34			51.8 ± 2.14			63.5 ± 14.04		
1.91E-07	22.3 ± 1.48			39.7 ± 2.16			**62.2 ± 12.89**		
9.54E-08	22.2 ± 2.58			52.8 ± 6.85			78.2 ± 14.62		
4.77E-08	30.7 ± 1			60.8 ± 11.03			88.9 ± 11.88		
2.38E-08	52.2 ± 10.47			58.8 ± 12.08			92.2 ± 19.61		
1.19E-08	60.6 ± 4.27			**67 ± 5.4**			82.1 ± 12.61		
5.96E-09	71.5 ± 3.17			77.9 ± 24.12			87.5 ± 16.06		
2.98E-09	**87.7 ± 5.81**			69.6 ± 8.48			101 ± 10.54		
1.49E-09	102.7 ± 3.47			81.4 ± 3.04			92 ± 6.8		
7.45E-10	101.8 ± 6.79			79.7 ± 0.66			98.7 ± 4.48		
3.73E-10	105.5 ± 2.4			88.8 ± 1.57			107.2 ± 15.64		
no drug	100 ± 2.97			100 ± 14.81			100 ± 11.67		
Voriconazole									
4	1.5 ± 0.4			3.6 ± 0.25			4.5 ± 0.64		
2	1.6 ± 0.34			4.1 ± 0.86			4.9 ± 0.95		
1	1.8 ± 0.33			4.3 ± 0.88			5.8 ± 1.72		
0.5	1.8 ± 0.58			6.7 ± 1.56			5.5 ± 1.11		
0.25	1.6 ± 0.41			10.6 ± 2.25			5.7 ± 1.01		
0.125	4 ± 1.36			28.7 ± 5.67			19.6 ± 1.97		
0.063	**21.6 ± 5.02**			**54.2 ± 2.46**			**81 ± 0.78**		
0.031	91.7 ± 1.29			100 ± 15.94			93 ± 4.35		
0.016	113.3 ± 10.33			104.2 ± 10.01			98.7 ± 5.49		
0.008	100.3 ± 3.63			103.6 ± 12.42			97.7 ± 10.31		
no drug	100 ± 7.41			100 ± 23.28			100 ± 10.05		

^*a*^ Strains were grown for 12 h at 37°C before microscopy-assisted growth analysis. Bold, the lowest concentration of each drug that significantly inhibited the growth of the individual species (*P* < 0.05). SD, standard deviation.

In previous work, the inhibitory potential of olorofim against planktonic cells of *A. fumigatus* isolates at sub-MIC levels and different time points (4 h, 12 h, 24 h, and 48 h) has already been demonstrated (13), however at a comparably low concentration range (up to 3 serial dilutions below the MIC). Here, we monitored and demonstrated its potent anti-*Aspergillus* activity using an extended sub-MIC range (up to 27 serial dilutions below the MIC), which allowed us to determine the smallest concentration that led to significant growth inhibition of each *Aspergillus* species tested. In contrast to the first-line treatment agent voriconazole, the *in vitro* activity of which was restricted to a relatively small concentration range for the *Aspergillus* spp. tested in this work (2–4 serial dilutions below the MIC at 48 h), at 12 h olorofim significantly inhibited the growth of *A. fumigatus*, *A. flavus*, and *A. niger*, respectively, 23 (8,388,608-fold), 21 (2,097,152-fold), and 18 (262,144-fold) serial dilutions below the finally detected MIC at 48 h. The low olorofim concentrations that were required to almost fully inhibit growth of the different *Aspergillus* species (>90%) at 12 h re-enforce the requirement of hyphae for pyrimidines to facilitate active growth.

The observed sub-MIC effects suggest that olorofim may continue to exert a growth inhibitory effect at levels below established MIC thresholds. This and the previously observed post antifungal effects (14) are helpful properties of olorofim as a new antifungal agent and suggest, like previous work (13), that olorofim might have antifungal effects also at low doses if drug levels fall below targeted therapeutic concentrations during standard dosing. However, it is important to note that population pharmacokinetic modeling (in-house data) has predicted that, with the standard dosing regimen, ≥94% of patients with invasive fungal infection (IFI) will have plasma concentrations above the therapeutic threshold for 24 h a day, with ≥98% of patients exceeding the therapeutic threshold for over 20 h a day.

Sub-MIC concentrations of antimicrobials are associated with an increased risk of resistance, particularly for antibacterials. Previously, exposure to sub-MIC concentrations of olorofim during serial passage experiments did not give rise to increased MICs after 40 passages (8). In contrast, there were significant increases in MICs of voriconazole after ~15 passages. Currently, on treatment, resistance to olorofim has not been seen clinically, although only a small number of IFI patients (*n* = 203) have been fully evaluated to date.

## SUPPLEMENTAL MATERIAL

Supplemental data 1 spectrum.03304-23-s0001.xlsx Supplemental data, including raw data that were used to calculate percent growth inhibition.

ASM does not own the copyrights to Supplemental Material that may be linked to, or accessed through, an article. The authors have granted ASM a non-exclusive, world-wide license to publish the Supplemental Material files. Please contact the corresponding author directly for reuse.

## References

[1] GAFFI. 2023. Fungal disease frequency. Available from: https://www.gaffi.org/why/fungal-disease-frequency

[2] Ullmann AJ, Aguado JM, Arikan-Akdagli S, Denning DW, Groll AH, Lagrou K, Lass-Flörl C, Lewis RE, Munoz P, Verweij PE, et al.. 2018. Diagnosis and management of Aspergillus diseases: executive summary of the 2017 ESCMID-ECMM-ERS guideline. Clin Microbiol Infect 24 Suppl 1:e1–e38. doi:10.1016/j.cmi.2018.01.002 29544767

[3] Patterson TF, Thompson GR, Denning DW, Fishman JA, Hadley S, Herbrecht R, Kontoyiannis DP, Marr KA, Morrison VA, Nguyen MH, Segal BH, Steinbach WJ, Stevens DA, Walsh TJ, Wingard JR, Young J-A, Bennett JE. 2016. Practice guidelines for the diagnosis and management of Aspergillosis: 2016 update by the infectious diseases society of America. Clin Infect Dis 63:433–442. doi:10.1093/cid/ciw444 27481947 PMC4967611

[4] Verweij PE, Chowdhary A, Melchers WJG, Meis JF. 2016. Azole resistance in Aspergillus fumigatus: can we retain the clinical use of mold-active antifungal azoles. Clin Infect Dis 62:362–368. doi:10.1093/cid/civ885 26486705 PMC4706635

[5] Verweij PE, Lucas JA, Arendrup MC, Bowyer P, Brinkmann AJF, Denning DW, Dyer PS, Fisher MC, Geenen PL, Gisi U, Hermann D, Hoogendijk A, Kiers E, Lagrou K, Melchers WJG, Rhodes J, Rietveld AG, Schoustra SE, Stenzel K, Zwaan BJ, Fraaije BA. 2020. The one health problem of azole resistance in Aspergillus fumigatus: current insights and future research agenda. Fungal Biology Reviews 34:202–214. doi:10.1016/j.fbr.2020.10.003

[6] Osherov N, Kontoyiannis DP. 2017. The anti-Aspergillus drug pipeline: is the glass half full or empty. Med Mycol 55:118–124. doi:10.1093/mmy/myw060 27562862

[7] Gintjee TJ, Donnelley MA, Thompson GR. 2020. Aspiring antifungals: review of current antifungal pipeline developments. J Fungi (Basel) 6:28. doi:10.3390/jof6010028 32106450 PMC7151215

[8] Oliver JD, Sibley GEM, Beckmann N, Dobb KS, Slater MJ, McEntee L, du Pré S, Livermore J, Bromley MJ, Wiederhold NP, Hope WW, Kennedy AJ, Law D, Birch M. 2016. F901318 represents a novel class of antifungal drug that inhibits dihydroorotate dehydrogenase. Proc Natl Acad Sci USA 113:12809–12814. doi:10.1073/pnas.1608304113 27791100 PMC5111691

[9] Hoenigl M, Sprute R, Egger M, Arastehfar A, Cornely OA, Krause R, Lass-Flörl C, Prattes J, Spec A, Thompson GR, Wiederhold N, Jenks JD. 2021. The antifungal pipeline: fosmanogepix, Ibrexafungerp, olorofim, opelconazole, and rezafungin. Drugs 81:1703–1729. doi:10.1007/s40265-021-01611-0 34626339 PMC8501344

[10] Kirchhoff L, Dittmer S, Buer J, Rath PM, Steinmann J. 2020. In vitro activity of olorofim (F901318) against fungi of the genus, scedosporium and rasamsonia as well as against lomentospora prolificans, Exophiala dermatitidis and azole-resistant Aspergillus fumigatus. Int J Antimicrob Agents 56:106105. doi:10.1016/j.ijantimicag.2020.106105 32721601

[11] Wurster S, Kumaresan PR, Albert ND, Hauser PJ, Lewis RE, Kontoyiannis DP. 2019. Live monitoring and analysis of fungal growth, viability, and mycelial morphology using the incucyte neurotrack processing module. mBio 10:e00673-19. doi:10.1128/mBio.00673-19 31138745 PMC6538782

[12] Guinea J, Meletiadis J, Arikan-Akdagli S, Muehlethaler K, Kahlmeter G, Arendrup MC. 2022. EUCAST definitive document EDef 9.4: method for the determination of broth dilution minimum inhibitory concentrations of antifungal agents for conidia forming moulds. Available from: https://eucast.org/astoffungi/

[13] Kirchhoff L, Dittmer S, Furnica DT, Buer J, Steinmann E, Rath PM, Steinmann J. 2022. Inhibition of azole-resistant biofilm at various formation stages by antifungal drugs, including olorofim. J Antimicrob Chemother 77:1645–1654. doi:10.1093/jac/dkac062 35289361

[14] du Pré S, Beckmann N, Almeida MC, Sibley GEM, Law D, Brand AC, Birch M, Read ND, Oliver JD. 2018. Effect of the novel antifungal drug F901318 (Olorofim) on growth and viability of Aspergillus fumigatus. Antimicrob Agents Chemother 62:e00231-18. doi:10.1128/AAC.00231-18 29891595 PMC6105813

